# Acute Dengue Fever with Computed Tomography (CT) Correlation

**DOI:** 10.4269/ajtmh.2011.10-0710

**Published:** 2011-05-05

**Authors:** Helen C. Addley, Lawrence Green, Sophie Petitclerc

**Affiliations:** Department of Radiology, Montreal General Hospital, Montreal, QC, Canada; J. D. MacLean Centre for Tropical Disease, McGill University, Montreal, QC, Canada

**Figure 1.**
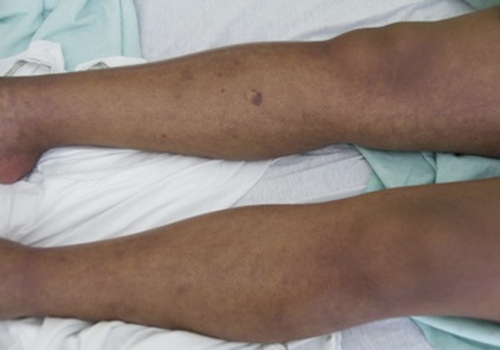
Purpuric rash on both legs. This figure appears in color at www.ajtmh.org.

A 23-year-old female presented to the emergency department with vomiting and abdominal pain. She had traveled home to Canada the previous day from Indonesia and Thailand. Her symptoms had started 5 days previously in Bangkok with malaise and fever. On clinical examination she looked ill with a low-grade fever of 37.3°C. Cardiovascular, respiratory, and neurological examinations were normal. On examination, her abdomen was extremely tender with guarding of the right upper quadrant. A diffuse purpuric erythematous rash was noted over her extremities and trunk (Figure 1).

**Figure 2.**
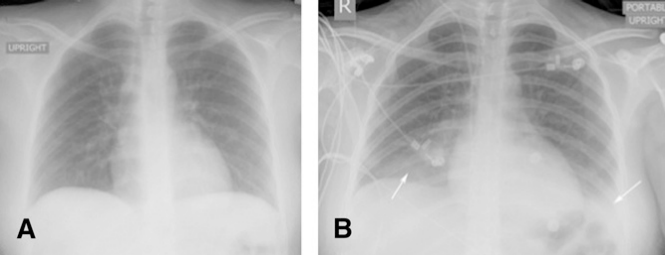
(**A**) Chest radiograph at initial presentation and (**B**) after 24 hours showing bilateral pleural effusions (white arrows).

Laboratory tests revealed a low platelet level at 34 with raised liver transaminases: aspartate aminotransferase 244 and alanine aminotransferase 111. Malaria smear was negative. Chest radiograph at initial presentation was negative but within 24 hours after the highest fever at 39.4°C showed moderate bilateral pleural effusions (Figure 2).

**Figure 3.**
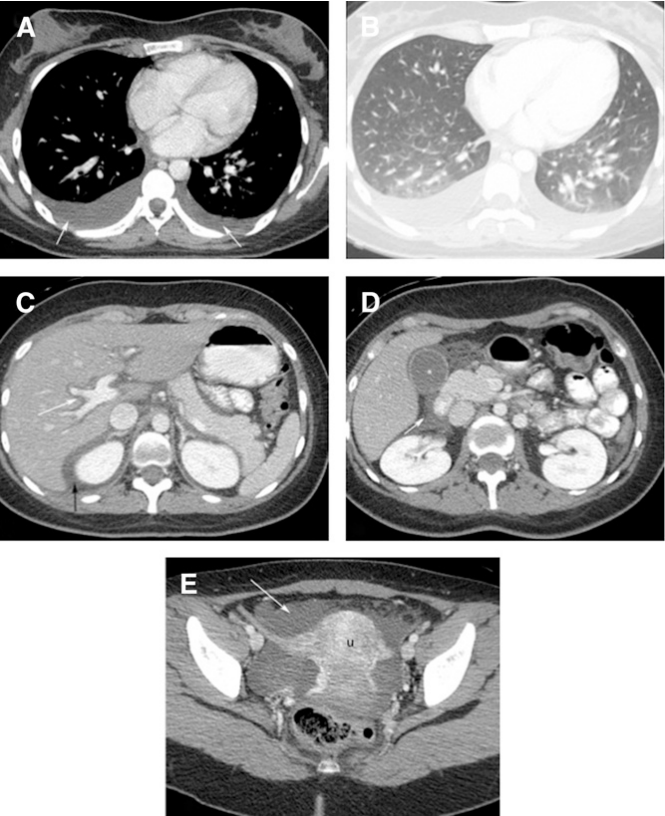
Computed tomography (CT) of the abdomen including the lung bases (**A**) showed bilateral pleural effusions without evidence of pulmonary edema on lung windows (**B**). Moderate amount of free fluid seen in the upper abdomen (**C**, **D**) with periportal edema (white arrow; **C**), and fluid in Morrison's pouch (black arrow; **C**). Pericholecystic fluid (white arrow; **D**) surrounding the gallbladder (* **D**) is shown. Intraperitoneal fluid is also seen in the pelvis (white arrow; **E**) outlining the uterus (u).

Computed tomography (CT) of the abdomen showed a moderate amount of low-attenuation free fluid in the abdomen and pelvis with periportal edema and pericholecystic fluid (Figure 3).

The combination of thrombocytopenia and imaging findings consistent with plasma leakage are suggestive of dengue hemorrhagic fever^1^; later confirmed on serology with both single IgG and IgM positive results. Imaging in assessment of fluid in multiple body compartments becomes detectable at the time of the immune response and typically at the time of disappearance of fever and is well demonstrated on both CT and ultrasound examinations.^2^
